# Anisotropic Carbon Nanotube Structures with High Aspect Ratio Nanopores for Li-Ion Battery Anodes

**DOI:** 10.1021/acsanm.1c01157

**Published:** 2021-06-16

**Authors:** Sarah Jessl, Simon Engelke, Davor Copic, Jeremy J. Baumberg, Michael De Volder

**Affiliations:** † Department of Engineering, University of Cambridge, Cambridge CB2 1PZ, United Kingdom; ‡ Cambridge Graphene Centre, University of Cambridge, Cambridge CB3 0FA, United Kingdom; § NanoPhotonics Centre, Cavendish Laboratory, University of Cambridge, Cambridge CB3 0HE, United Kingdom

**Keywords:** carbon nanotubes, colloidal lithography, chemical vapor deposition, high-aspect ratio structures, submicron pores

## Abstract

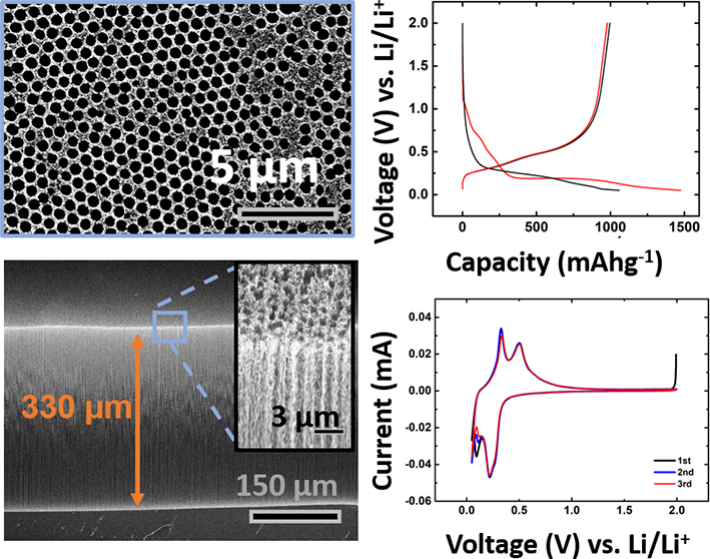

Technological advances in membrane technology, catalysis, and electrochemical energy storage require the fabrication of controlled pore structures at ever smaller length scales. It is therefore important to develop processes allowing for the fabrication of materials with controlled submicron porous structures. We propose a combination of colloidal lithography and chemical vapor deposition of carbon nanotubes to create continuous straight pores with diameters down to 100 nm in structures with thicknesses of more than 300 μm. These structures offer unique features, including continuous and parallel pores with aspect ratios in excess of 3000, a low pore tortuosity, good electrical conductivity, and electrochemical stability. We demonstrate that these structures can be used in Li-ion batteries by coating the carbon nanotubes with Si as an active anode material.

## Introduction

Continuous advances in nanotechnology require highly organized structures on the nano- and micron-scales for applications in advanced membranes,^1,2^ battery electrodes,^3−5^ catalyst supports,^6,7^ advanced manufacturing processes,^8^ and photonics,^9^ microelectronics, chemical and biological sensing, as well as bioengineering.^10^ Carbon nanotubes (CNTs) are interesting materials for applications such as water purification and filtration,^11−13^ electrodes for supercapacitors and batteries,^14−17^ and flexible electronics^18−20^ due to their unique mechanical, thermal, and electrical properties.^21,22^ Much research has focused on the synthesis of aligned CNT “forests”^23−25^ and their patterning with techniques such as UV lithography.^26^ However, lithography equipment for creating sub-500 nm patterns is expensive; therefore, this paper explores colloidal lithography as a versatile low-cost and large-area^27^ patterning method^28−31^ for CNTs and also compares it to interference lithography (IL) as an alternative patterning method. We demonstrate that this process can be used to pattern high-aspect ratio vertically aligned CNTs (VACNTs), resulting in submicron channels (diameters down to ∼100 nm) that are aligned perpendicularly to the substrate and with heights up to 330 μm. Patterning of CNT catalyst using colloidal lithography has been shown before by Mathur et al.^32^ for pillars of ∼10 μm and by Man et al.^33^ for lower height structures and their magnetic properties; this paper, however, focuses on the ability to form structures that reach aspect ratios of over 3000 and have the advantage of offering straight continuous pores without any internal dead-ends for a broad range of applications. Finally, we show how this process can be used for creating thick Li-ion battery (LIB) anodes with a good tortuosity. For this demonstration, we coated the VACNT structure with silicon, which is an alloy anode material pursued in both academic and industrial settings.^34−37^ We anticipate that this new electrode structure is interesting because of the high electric and thermal conductivity of the CNTs,^38^ as well as the efficient ion transport through the low tortuosity pores. Hybrid VACNT structures for energy applications have already been reported for plain forests^14,39−41^ or those prestructured into cones^20^ and pillars.^42,43^ However, to our knowledge, no attempts have been made to pattern CNT current collectors with such high-aspect ratio submicron pores. Finally, we compare the proposed colloidal lithography process with IL as a method for patterning nanopores in CNT forests and show a hydrothermal coating of iron oxide flakes as an alternative for chemical vapor deposition (CVD) of Si.

### Structure Fabrication Process

**Figure 1:**
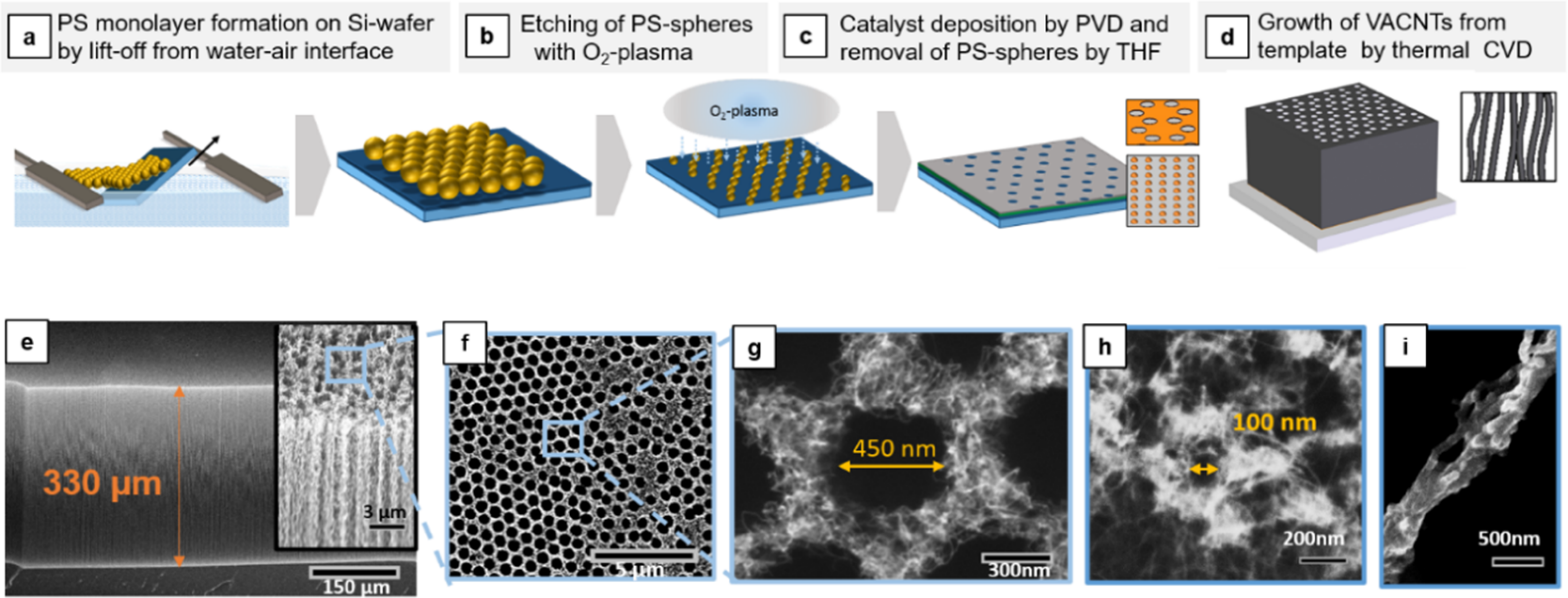
Step-wise fabrication process for the porous VACNT structure is shown here in the images (a)–(c) in the upper row; SEM images of the structure after (d) are shown in the bottom row: the side of a forest (e), the top (f) for a pore size of 450 nm (g) and 100 nm (h), including the coating with silicon (i) as an application example.

**Figure 2:**
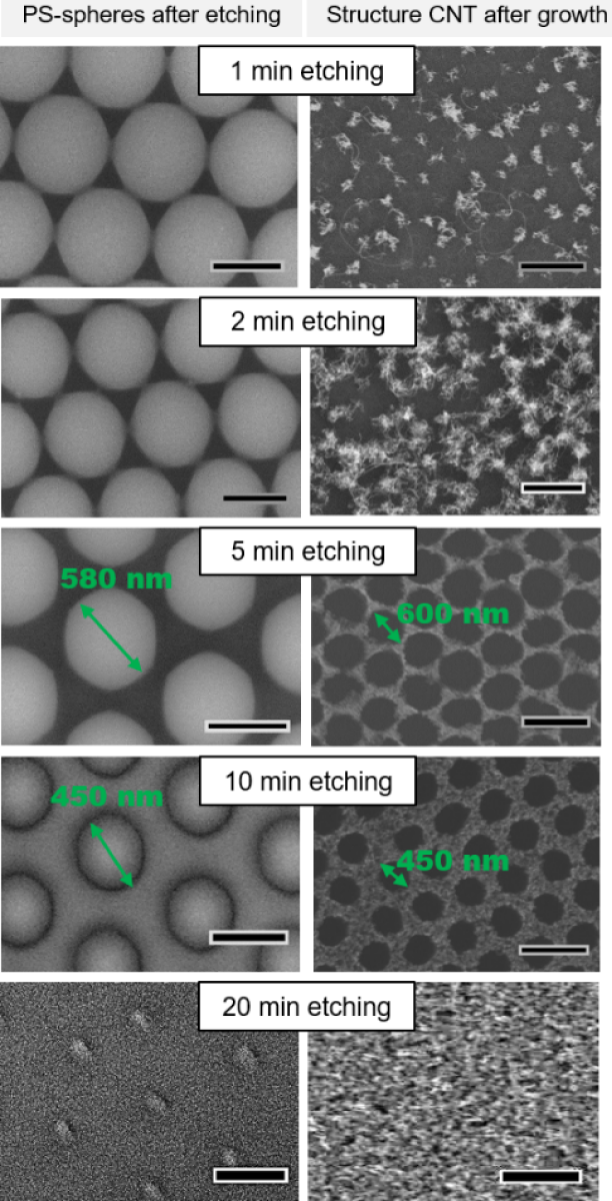
Left row shows a decrease in the size of the PS spheres with increasing O_2_ plasma time. The spheres with an etching time of 1 min have not changed much yet and are still connected by bridges, whereas the spheres after 20 min etching are barely visible anymore and have significantly shrunken in size. The right row shows the CNT forest structure. After 1 min, there is barely any CNT growth visible as the template did not have much space for catalyst deposition, whereas after 20 min, the holes by the spheres are so small that they are not visible in the forest anymore. Scale bars in left column are 500 nm, in right column, 1 μm.

The process for fabricating our high-aspect ratio VACNT–Si hybrid structures with submicron pores is depicted in Figure 1. First, we use colloidal lithography to template the catalyst layer monolayer of polystyrene (PS) spheres packed on a liquid–air interface using a version of the protocol described by Vogel et al.,^44^ adapted for working with a Langmuir–Blodgett trough instead of a Petri dish (Figure 1a). This results in a layer of hexagonally packed PS spheres, which is then transferred to a Si wafer as shown in Figure 1b, resulting in close-packed PS spheres with domain sizes of ∼10 μm over large areas (Figure S1). Next, O_2_ plasma is used to etch the PS spheres and thus to introduce a controlled gap between them (Figure 1b, Experimental Section). Note that simply changing the initial diameter of the PS spheres and the etching time provides good control over the pattern dimensions. Here, we used PS spheres with a diameter of 300 and 800 nm (though size scales from 50 to >1000 nm can potentially be used) and etching times of 1–20 min (see further, Figures 2 and S2).

Next, 10 nm of Al_2_O_3_ and 1 nm of Fe catalyst for VACNT growth are deposited on the substrate by electron-beam evaporation (see Experimental Section). Subsequently, this layer is pattered using a lift-off process where the PS spheres are dissolved in tetrahydrofuran (THF). After washing and drying, this results in a patterned catalyst layer ready for VACNT growth (Figure 1c). The VACNTs are synthesized using an atmospheric pressure CVD process (this is a well-established process;^23,45^ see Experimental Section, Figure 1d). By adjusting the growth time and temperature, the height of the structure and, thus, the aspect ratio can be tuned. By adjusting the etching time, the pore size can be tuned (Figure 1g,h). We also demonstrate the versatility in the surface chemistry by coating the structures with silicon (Figure 1i) and iron oxide.

## Results and Discussion

Colloidal lithography is used here for the first time as an easy and scalable method to produce large area VACNTs with nanoscale patterns (Figure 1). These structures are uniform over large areas (Figure S2a) and can be grown to a height of up to 330 μm (Figure S2b), thus creating pores with aspect ratios of more than 700 when 800 nm PS spheres with an etching time of 10 min are used (Figure S2c). These vertically aligned pores have diameters of ∼450 nm and a wall thicknesses of ∼230 nm. Further, monolayers using 300 nm PS spheres can achieve aspect ratios up to 3000 due to smaller pores of about 100 nm (see Figure S3). Overall, this process results in a dual porosity with pores that are hundreds of nanometers, which are defined by colloidal lithography and a smaller pore size of tens of nanometers that result from the space between the CNTs during the CVD synthesis.

The influence of etching times on the size of the 800 nm PS particles is shown in Figure 2. The left column shows the decrease of the PS sphere size with increasing etching time. After 1 min of O_2_ plasma etching, the spheres are still connected, resulting in triangular catalyst patches, which do not yield VACNTs (see right column) because they are mechanically unstable. Increasing the etching time leads to increasingly thick VACNT walls and aligned self-supported VACNT growth. A comparison between 5 and 10 min etching time can also be found in Figure S4.

**Figure 3:**
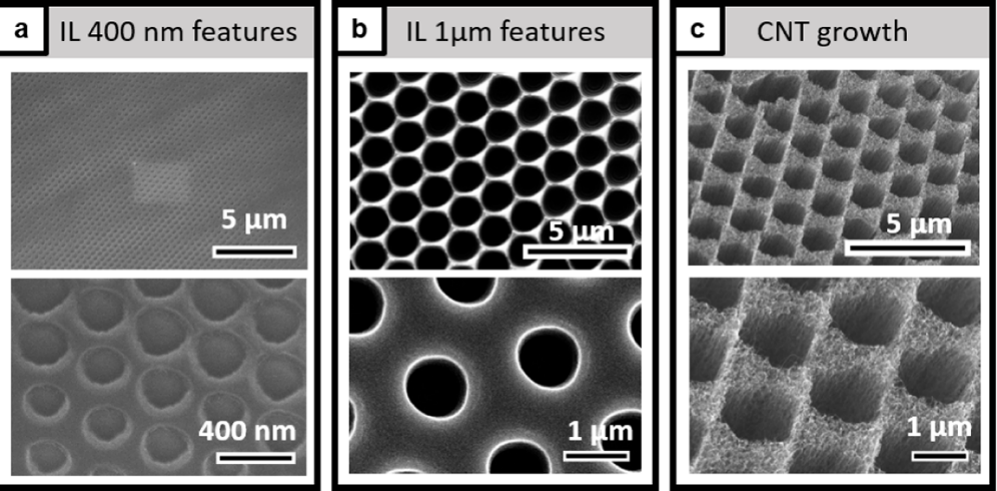
SEM images showing the IL pattern before growth for 400 nm features (a), for 1 μm features (b), and after CNT growth for 1 μm features (c).

As mentioned earlier, an alternative approach to achieve periodic features of submicron scale is laser IL. This process is a mask-free photolithographic technique in which interference patterns are generated by two laser beams and are printed directly into a photoresist.^46^ Compared to colloidal lithography, this process does not rely on the formation of a defect-free monolayer and subsequent etching but can easily be repeated once the laser configurations are optimized for a specific pattern. Results of patterning by IL and subsequent CNT growth are shown in Figure 3. Two different settings for the laser beams were used, resulting in 400 nm features (a) and 1 μm features (b, larger spacing). Figure 3c shows CNTs grown from the larger pattern. Simple, nonvacuum IL can easily reach features down to 400 nm, which was why we selected this size and a larger one for comparison. Fabricating smaller features requires a more complicated setup and was not explored further in this work.

The results above demonstrate that by changing the size and the etching time of the PS spheres or IL parameters, it is possible to create a wide range of pore sizes depending on the desired application. The aspect ratio of the pores can be tuned simply by changing the CNT synthesis time. Many applications using CNTs require some additional material to be deposited on their surface to extend their properties. However, most CNT coating methods are developed for CNT powders, whereas the functionalization of CNT forests is still facing challenges such as the detachment of the CNTs from the substrate and unwanted changes of the CNT alignment due to capillary aggregation when using wet chemistry.^47^ For this reason, gas-phase coating methods such as CVD and ALD are most commonly used for CNT forest modification.^48−51^ To demonstrate the versatility of the above colloidally patterned CNTs, we show both a dry CVD Si coating and a wet hydrothermal Fe_2_O_3_ coating process.

**Figure 4:**
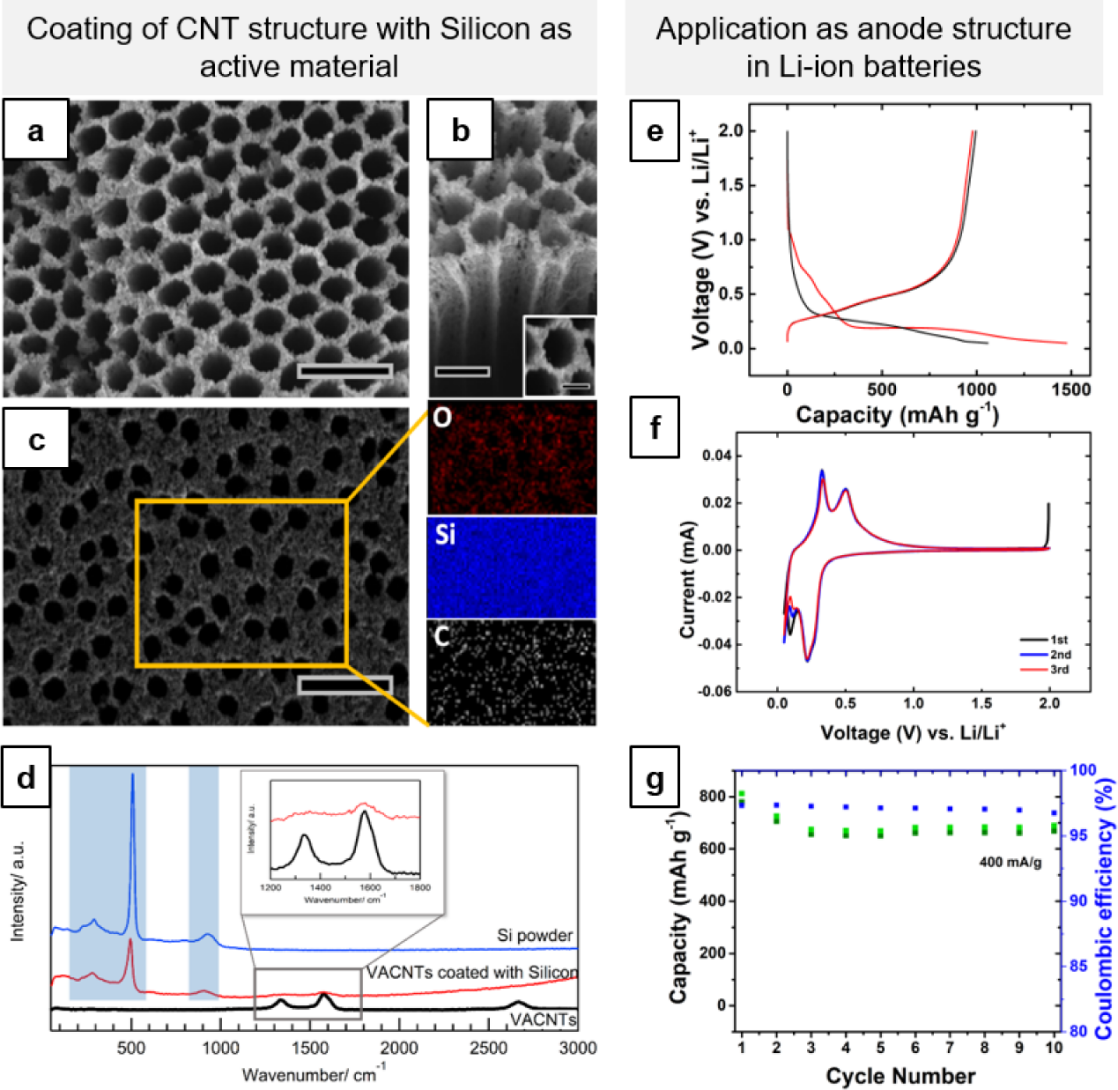
(a) Three-dimensional CNT structure (made by colloidal lithography) coated with Si by a thermal CVD process. (b) Tilted view of the Si-coated side walls. (c) SEM image and EDX mapping of a Si-coated CNT structure. (d) Raman spectra before and after Si coating. (e) Initial two galvanostatic charge/discharge curves of a Si-coated CNT structure (∼100 mA/g), (f) CV curves (0.01 mV/s) with peaks corresponding to the (de)lithiation of amorphous silicon, and (g) capacity retention over 10 cycles after the two formation cycles shown in Figure 4e (∼400 mA/g). Scale bars are 2 μm in (a), 1 μm in (b) and 500 nm in the inset, and 2 μm in (c).

Figure 4a shows a Si CVD-coated structure produced with the colloidal patterning process. Depending on the time and temperature of this Si CVD process, the thickness of the coating can be controlled (Figure S5). EDX mapping of the top surface (Figure 4c) and the bottom of the electrode (after the transfer printing, see Figure S6) shows that Si coated all the way through the VACANT internal structure, though the top surface has a thicker coating. Figure 4d shows the Raman spectra of VACNTs before and after the Si CVD coating along with that of silicon powder. The silicon peaks are clearly visible in the coated sample while the characteristic D and G peaks between 1300 and 1700 cm^–1^ are suppressed after coating (see inset). This suggests a good coating of the CNT surface with silicon after the CVD process.

The colloidal lithography-patterned VACNTs can also survive a microwave-assisted hydrothermal coating method (see Figure S7), for which we adapted a protocol reported by Li et al.^52^ and Jessl et al.^53^ to coat CNTs with Fe_2_O_3_ nanosheets. While this method worked for short structures (as seen in the SEM images in Figure S7a,b), it is more difficult for higher aspect ratio structures, as the narrow pores tend to close during drying due to capillary forces (as seen in the SEM images in Figure S7c,d).

We anticipate that the ability to structure CNTs with controlled nanoscale-aligned pores as well as controlled material composition can find applications in a range of domains, including catalysis, water filtration, and electrochemical energy storage. Here we show that the silicon-coated structures can be used as anodes in LIBs (Figure 4e–g). The change in capacity with an increase in rate and cycles can be found in Figure S9. The advantage of the CNT scaffolds is that they offer a good electrically conductive network, the straight pores offer good ion transport with a tortuosity of 1,^41,54^ and the CNT structures can act as a buffer for volume change and aggregation.^55^ Finally, silicon is a promising alloy-type anode material^34,56^ for high-energy density LIBs because its theoretical specific capacity is more than 10 times higher than that of the commercially used graphite (4200 mAh/g).^34^ Silicon can be deposited via the CVD route^57,58^ and has been reported by Gohier et al.^59^ on unpatterned CNT forests and as a combination of catalyst-free CNTs with Si nanowires by Harpak et al.^60^ for battery applications.

In the electrochemical experiments presented here, the height of the electrode used is 150 μm, which is about three times thicker than classic battery electrodes.^61,62^ The initial pore diameter was 400–500 nm before coating with Si. The electrode is then transfer printed on a Cu film using a process described previously^20^ and measured in a half-cell configuration. During the transfer process, the electrodes are slightly compressed (see Figure S8**)** but the temperature treatment step of the current collector does not introduce any changes to the material (see Figure S8d where the oxygen amount is shown to not change the oxygen amount significantly, neither on the top nor at the bottom of the sample).

The electrode shows a first cycle coulombic efficiency of ∼77% (Figure 4e) and a capacity of about 1000 mAh/g. This is lower than the theoretical capacity, which might be due to our conservative estimate of the Si coating weight, which is difficult to measure accurately, and due to poor utilization of the active material in these ultrathick electrodes. The cyclic voltammetry (CV) curves (0.01 mV/s) show two sharp peaks during lithiation (Figure 4f). The first can be assigned to the high-voltage lithiation of amorphous silicon at ∼0.25 V and the second peak to the low-voltage lithiation of amorphous silicon near 0 V. During the delithiation process, two peaks corresponding to the sequential extraction of the Li-ions (a-Li*_x_*_′+*x*″_Si → a-Li*_x_*_′_Si + *x*″Li → a-Si + *x*′Li) are found at 0.3 and 0.5 V. The shape of this curve is consistent with that in the previous reports of amorphous silicon.^42,63,64^ As shown in Figure 4g, the capacity retention over 10 cycles (after the two formation cycles shown in Figure 4e) remains stable at ∼650 mA/hg. These experiments provide a first indication that the proposed porous CNT structures might be a good scaffold for LIB electrodes, and we anticipate that because of the tuneability of the pore size and surface chemistry, they may also be attractive for a number of other applications such as filtration^45,65,66^ and catalysts.^6,7^ The implementation of this system into real-life batteries, however, will require much more investigation and optimization.

## Conclusions

In conclusion, we show a process to fabricate VACNT structures with submicron pores using colloidal and interference lithographies. With these manufacturing processes, we demonstrate a cost-effective, flexible, and scalable approach to pattern CNT forests. Using this process, pores with high-aspect ratios of up to 3000 can be achieved and the pore size as well as the thickness of the walls made out of CNTs can be adjusted to control the dimensions of the resulting structure. Additionally, we have shown that it is possible to coat these patterned VACNT structures with dry (CVD) and wet (hydrothermal) processes. CVD was used to coat the VA–CNT structures with silicon, thus creating a hybrid structure that can be used as a thick LIB anode. We anticipate that these hybrid materials with controlled pore structures will also find applications in other nanotech domains, including membrane technology, catalysis, and water filtration.

## Experimental Section

### Colloidal Monolayer Formation on Si Wafer

The catalyst layer is patterned using colloidal lithography. For this process, a monolayer of PS spheres (Polybead Microspheres 0.8 and 0.3 μm) is deposited on a silicon wafer using the method reported by Vogel et al.^44^ combined with a Langmuir–Blodgett trough (KSV-Nima). The PS spheres were deposited on the surface of a 0.1 mM sodium dodecyl sulfate (SDS, Sigma Aldrich; 288.372 g/mol) in water solution using a micromanipulator. The pH of the SDS solution is adjusted to 11 by addition of ammonium hydroxide (NH_4_OH, Fisher Scientific, 30%, 35.046 g/mol). Then the barriers of the Langmuir–Blodgett trough are moved together until a uniform, closely packed monolayer on the surface is achieved, which is then transferred onto a Si wafer. This monolayer is dried at a 45° angle. The prepared monolayers are then etched with O_2_ plasma (0.8 mbar, 50% O_2_, Diener Femto Plasma etcher (60 Hz/ 3680W)) for various times to decrease the size of the PS spheres (see Figure 2).

### Interference Lithography

The laser IL is done following a paper by Brooks et al.^46^ To summarize, after depositing a 2 μm layer of resist (AZ 5214 E) onto the Si substrate by spin-coating, the substrate is patterned using a pair of ultraviolet (405 nm) laser beams. A fringe pattern is generated with a grating constant of 400 nm and 1 μm by varying the angles of the lasers. A sequence of three exposures is employed to create the pattern, with a 60° rotation of the sample between illuminations before developing the photoresist.

### Patterning of Catalyst for CNT Synthesis

After the O_2_ plasma etching step, e-beam physical vapor deposition is used to deposit the catalyst (EV-Lesker, PVD 75, four-crucible e-beam evaporator). The catalyst layer consists of a 10 nm alumina layer (Al_2_O_3_, 1 Å/s), followed by 1 nm iron layer (Fe, 0.25 Å/s). Afterward, the PS spheres are dissolved by sonication in THF (Fisher Scientific, 72.107 g/mol) for 20 min at 40 °C and a cleaning step of 3 min sonication in isopropanol (IPA, Fisher Scientific, 60.096 g/mol), leaving a hexagonally ordered hole structure on the substrate.

### CNT Synthesis

For the synthesis of VACNTs, an atmospheric pressure tube furnace is used. The samples are grown using a standardized procedure with a catalyst annealing time of 15 min under a 100 sccm helium and 400 sccm hydrogen gas stream. Subsequently, ethylene gas is introduced at 100 sccm to start the CNT growth. The VACNT growth height can be controlled by the temperature and growth time. Here, the CNTs are grown at 720 °C for 16 min. After the growth time, the tube is pulled out of the furnace allowing the substrates to cool down for 2 min while the helium–hydrogen–ethylene gas stream is still flowing.

### Silicon Deposition by Thermal CVD

The active material, silicon, is deposited on the structure using a thermal CVD system and a published process for Si nanowires.^57,58^ SiH_4_ as a precursor is used in an argon atmosphere to achieve the modification of the 3D CNT structures fabricated by colloidal lithography. The deposition is run at 550 °C for 15 and 30 min at 15 mbar.

### Fe_2_O_3_ Deposition by Wet Chemistry

For the synthesis of iron oxide (Fe_2_O_3_) on the VACNT structures, a published protocol for porous α-Fe_2_O_3_ nanosheets is modified for the use as a microwave-assisted synthesis.^52^ About 2 mM of iron(III) sulfate hydrate (Fe_2_(SO_4_)_3_·H_2_O, 244.98 g/mol, Fisher) and 8 mM of urea are dissolved in 50 mL of ethylene glycol (EG, 62.07 g/mol, Fluka). Polyvinylpyrrolidone (0.1 g) (PVP, K30, 40 000 g/mol, Fluka) is added to the solution. After stirring for 10 min, the solution is filled into Teflon liners for the microwave reactor (Multiwave Pro, Anton Paar). The VACNT structures on the Si-wafer chips are treated with air plasma for 10 min to increase the number of oxygen-containing surface groups and then slowly put into the solution. The liners are put into the microwave and heated to 160 °C for 5 h. Afterward, the solution is filtered using vacuum filtration and the CNT structure is washed multiple times with DI water. The filtered particles and the coated structure are dried at 80 °C overnight.

### Battery Fabrication and Electrochemical Measurements

CR 2032 type cells were assembled in an Ar-filled (<0.5 ppm H_2_O and <0.5 O_2_) glove box (MBraun). Coated anisotropic porous CNT structures and a pure Li metal foil were used as electrodes with 1.0 m LiPF_6_ ethylene carbonate/dimethyl carbonate (Sigma Aldrich, battery grade) as electrolyte and a Celgard separator. The electrochemical measurements were carried out using a VMP3 Biologics multichannel potentiostat/battery cycler.

## Supporting Information Available

Additional figures as referenced in the main manuscript. SEM of large area overview of the PS monolayer, SEM images showing monolayer of 300 nm PS spheres, direct comparison of pore and wall sizes, EDX mapping of the top of the coated structure, coating of VACNT structures patterned by colloidal lithography, Rate test for showing the capacity vs. cycle number (PDF)
