# Supplementary material for: Anisotropic Carbon Nanotube Structures with High Aspect Ratio Nanopores for Li-Ion Battery Anodes
Source: ACS Appl Nano Mater. 2021 Jun 16;4(6):6299–305. doi: 10.1021/acsanm.1c01157 (PMC8240089; doi:10.1021/acsanm.1c01157)
Supplement: Supplementary file 1 — an1c01157_si_001.pdf [file an1c01157_si_001.pdf]

Supporting Information:

Anisotropic Carbon Nanotube Structures with High Aspect Ratio Nanopores for Li-Ion Battery Anodes

Author(s), and Corresponding Author(s): Sarah Jessl[1], Simon Engelke[1,2], Davor Copic[1], Jeremy J. Baumberg[3] Michael De Volder[1,*]

[1] University of Cambridge, Department of Engineering, Cambridge CB2 1PZ, United Kingdom

[2] Cambridge Graphene Centre, University of Cambridge, Cambridge, CB3 0FA, United Kingdom

[3] NanoPhotonics Centre, Cavendish Laboratory, University of Cambridge, Cambridge CB3 0HE, United Kingdom

[*] Corresponding author. Tel: 01223 338176 E-mail: mfld2@cam.ac.uk

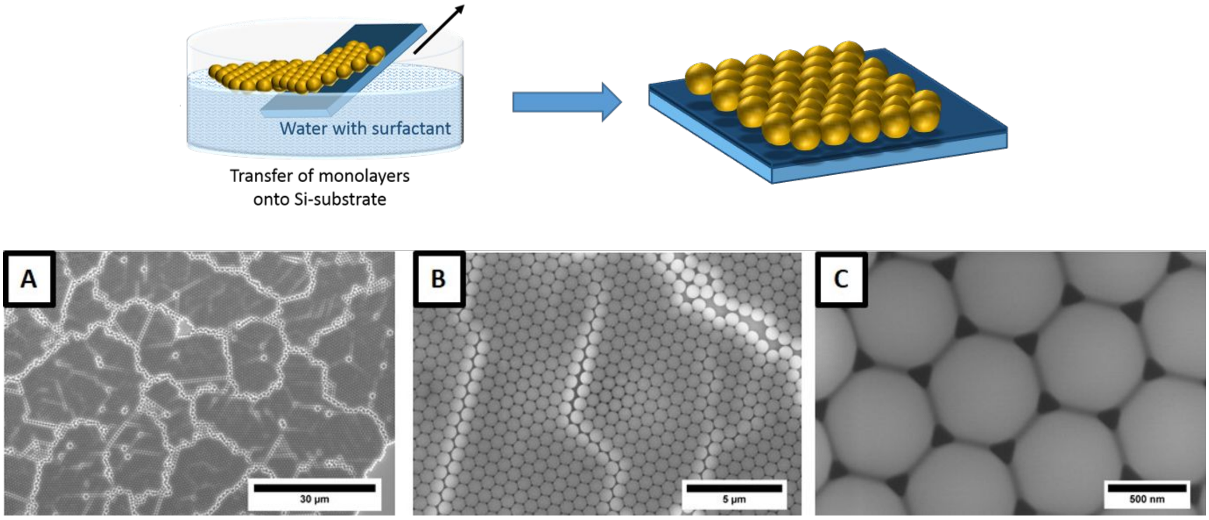

Figure S1: SEM of large area overview of the PS monolayer (A), higher magnification showing the hexagonal packing (B) and SEM of the 800 nm particles (C).

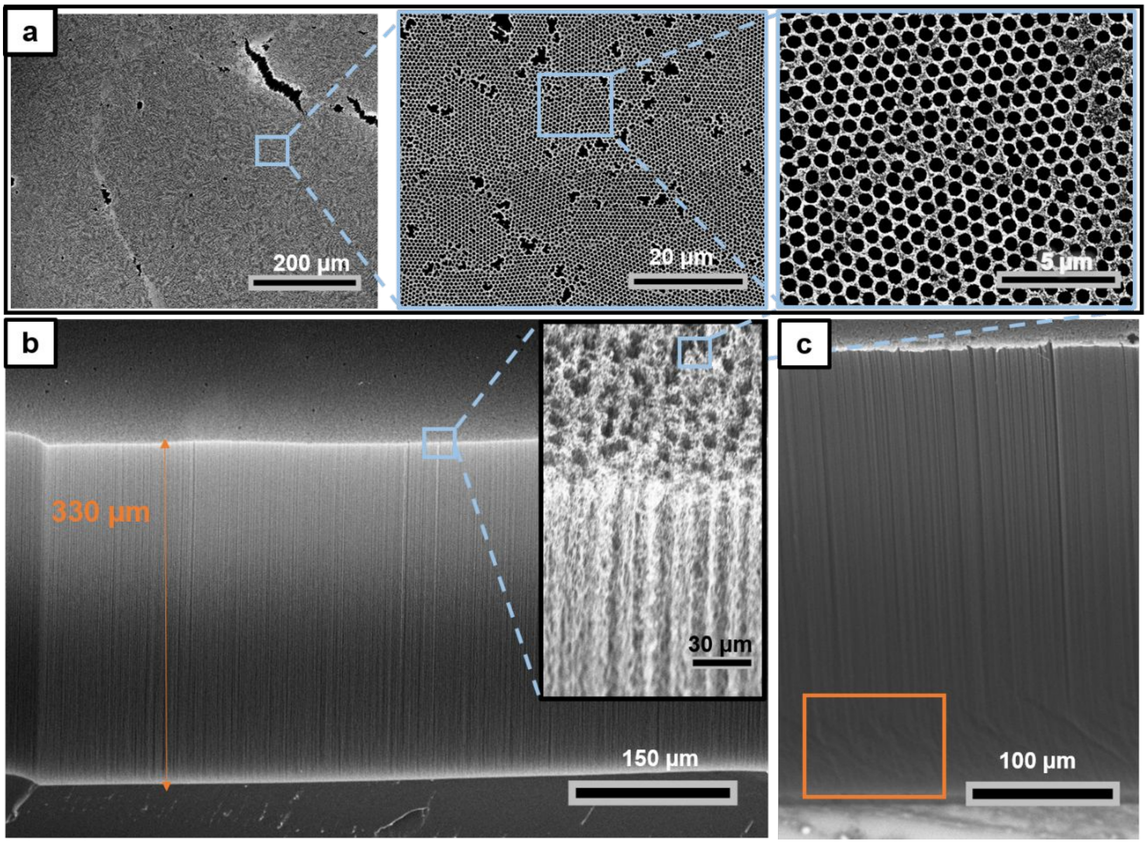

Figure S2: The SEM images in (a) show the large area that can be covered by the structure with 450 nm holes on several levels of magnification, in (b) the height of the forest from the colloidal structures is shown. The forest in (c) shows a possible failure mode in this growth where the structure slightly collapses with increasing height (see orange square).

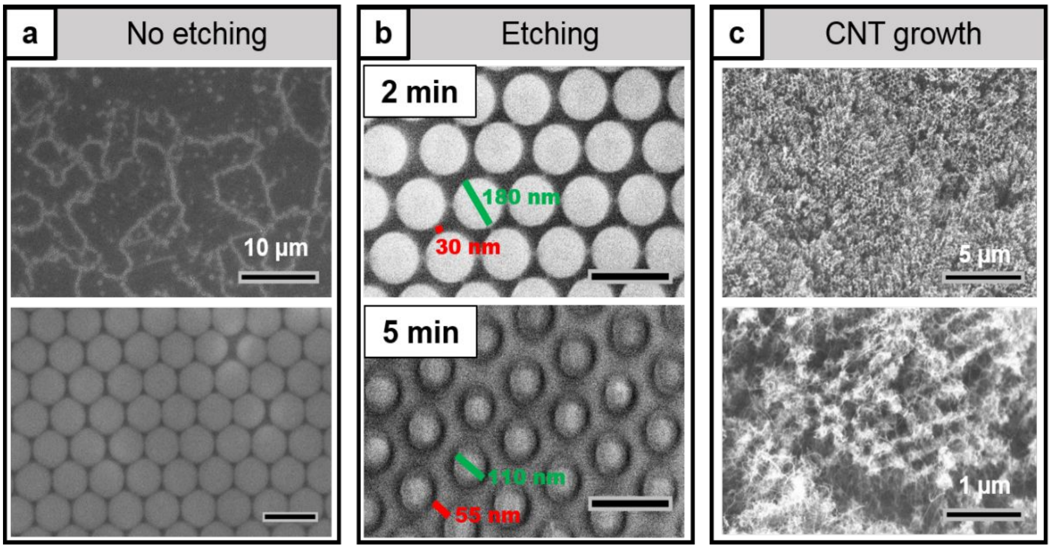

Figure S3: SEM images showing monolayer of 300nm PS spheres (a) without etching, (b) with different etching times with O_2-plasma and (c) after CNT growth.

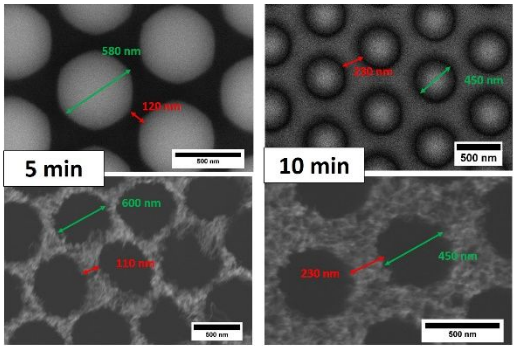

Figure S4: Direct comparison of pore and wall sizes for a 5 minute and 10 minute etching time.

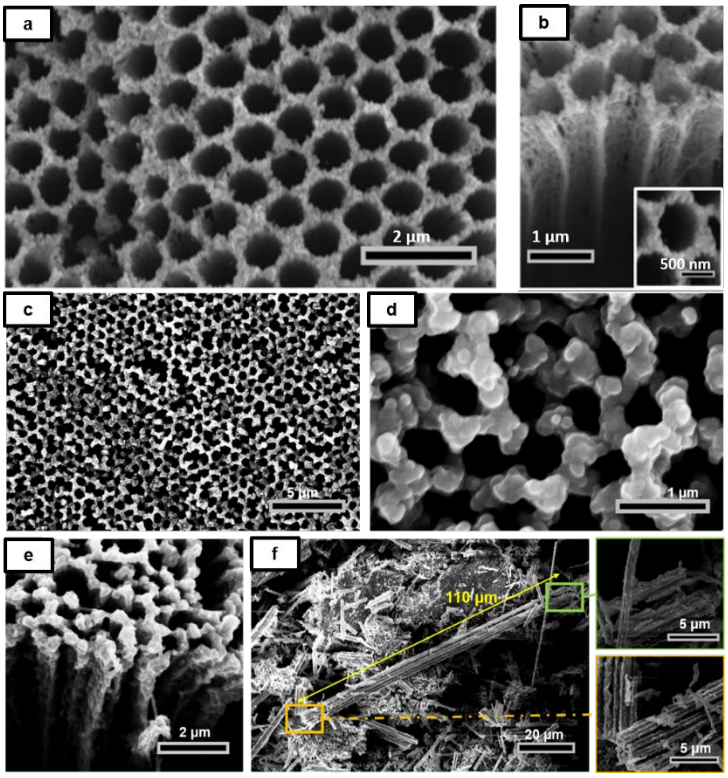

Figure S5: A thin coating with CVD-grown Silicon can be seen in (a)-(b). A thicker coating by an increase in coating time on the 3D CNT structure with nanoscale pores can be seen in (c)-(e). The transfer of this coated structure is attempted but it leads to breaking and incomplete transfer of it onto the Cu-tape (f). The adhesion to the substrate has significantly increased during the longer coating time. The long, coated CNT strand with the two higher magnification images at the top and bottom of the strand indicates that a good coating all the way through the pores could be achieved.

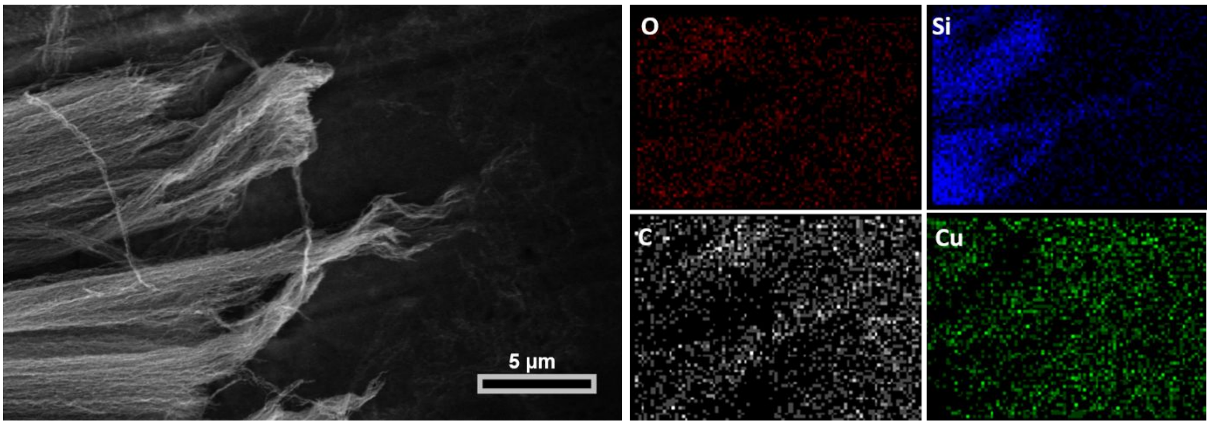

Figure S6: EDX mapping of the top of the coated structure after the transfer onto the copper tape, turning it into the bottom in contact with the copper tape.

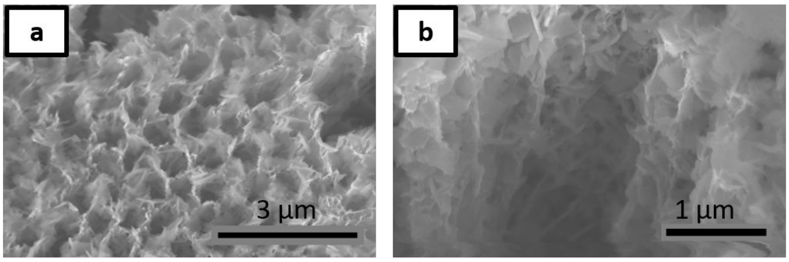

Figure S7: Coating of VACNT structures patterned by colloidal lithography with Fe2O3 from the top in (a) and and the side walls in (b), following a protocol by Li et al.[52]

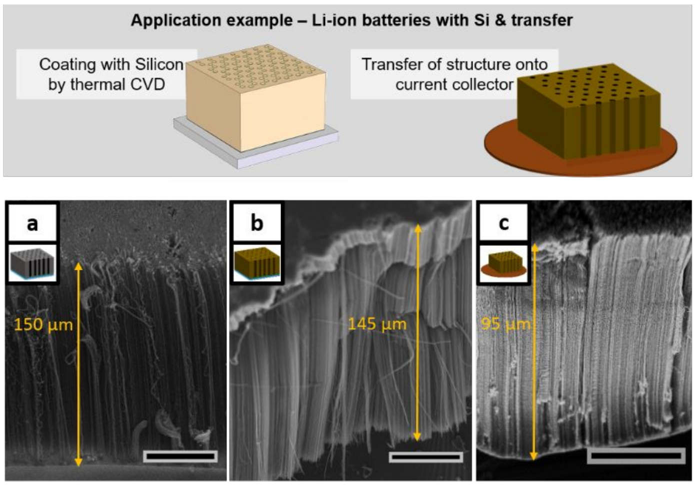

d

Atomic%	Before annealing	After annealing	
		Top	bottom
Si	94.88	88.31	85.56
O	5.12	11.69	14.45

Figure S8: While only a small change in height from 150 µm (a) to 145 µm (b) is seen during the modification, the transfer decreases the height of the coated 3D CNT structure to about 2/3 (c). EDX measurements before and after annealing showing no significant increase in the amount of oxygen (d). Scale bars are 50 µm.

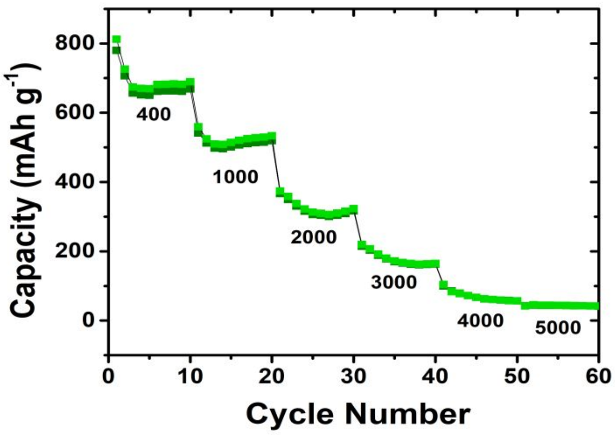

Figure S9: Rate test for showing the capacity vs. cycle number at increasing current density indicated in mA/g (400 mA/g to 5000 mA/g).
